# Factors affecting the ability of patients with complex vascular anomalies to navigate the healthcare system

**DOI:** 10.1186/s13023-024-03018-y

**Published:** 2024-01-18

**Authors:** Bryan Sisk, Sunny Lin, Anna M. Kerr

**Affiliations:** 1 Division of Hematology/Oncology, Department of Pediatrics, Washington University School of Medicine, St. Louis, MO USA; 2 Bioethics Research Center, Department of Medicine, Washington University School of Medicine, St. Louis, MO USA; 3 Informatics Institute, Department of Medicine, Washington University School of Medicine, St. Louis, MO USA; 4 Department of Primary Care, Heritage College of Osteopathic Medicine, Ohio University, Athens, OH USA

**Keywords:** Vascular malformation, Vascular anomaly, Health Care Access, Communication, Rare disease

## Abstract

**Background:**

Vascular anomalies (VAs) are rare congenital disorders that can cause pain, disfigurement, coagulopathy, asymmetric growth, and disability. Patients with complex VAs experience multiple barriers to accessing expert care. It is imperative to understand which factors support these patients’ ability to navigate the healthcare system.

**Results:**

We surveyed adult patients with VAs using previously validated measures, recruiting participants from five patient advocacy groups and multidisciplinary VA clinics. The primary outcome was self-reported ability to access needed medical care, using the “Navigating the Healthcare System” subscale of the Health Literacy Questionnaire. We evaluated factors associated with the ability to navigate the healthcare system using multivariate linear regression (*n* = 136). We also performed an exploratory model that included the primary care doctor’s knowledge of VAs for the subset of participants with a primary care doctor (*n* = 114). Participants were predominantly women (*n* = 90, 66%), White and non-Hispanic (*n* = 109, 73%), and college-educated (*n* = 101, 73%). Most participants had PIK3CA-Related Overgrowth Spectrum (*n* = 107, 78%). Most participants reported that navigating the healthcare system was “sometimes” or “usually difficult” (mean score 16.4/30, standard deviation 5.6). In multivariate linear regression, ability to navigate the healthcare system was associated positively with quality of information exchange (β = 0.38, 95% Confidence Interval (CI) 0.22 to 0.55, *p* <.001) and whether patients had VA specialists (β = 2.31, 95% CI 0.35 to 4.28, *p* =.021), but not associated with patient self-advocacy, anxiety, education, age, race and ethnicity, gender, or having a primary care doctor. In exploratory analysis of participants with primary care doctors, ability to navigate the healthcare system was positively associated with quality of information exchange (β = 0.27, 95% CI 0.09 to 0.45, *p* =.004), having a VA specialist (β = 2.31, 95% CI 0.22 to 4.39, *p* =.031), and primary care doctors’ VA knowledge (β = 0.27, 95% CI 0.04 to 0.50, *p* =.023).

**Conclusion:**

Patients with VAs struggle to navigate the healthcare system. High-quality information from clinicians and more knowledgeable primary care doctors might help patients to access needed care. Relying on patient self-advocacy is insufficient. Future efforts should focus on patient-directed and clinician-directed educational interventions. Additionally, future work should assess the structural barriers that impede healthcare access for these patients.

## Introduction

Vascular anomalies (VAs) are rare congenital disorders that can cause pain, disfigurement, coagulopathy, asymmetric overgrowth, disability, and stigma [1–6]. VAs include both vascular tumors and vascular malformations that develop in utero [2]. The presentation, symptoms, and extent of VAs can vary widely—some patients have small, isolated VAs that may resolve without intervention, and others have complex VAs that involve other major organs or parts of the body and require prompt evaluation and treatment [7–9]. VAs are most commonly driven by somatic mutations in genes in the mammalian target of rapamycin (mTOR) pathway (such as PIK3CA and TEK) or the RAS pathway (such as MAP2K1 and KRAS) [10–14]. Additionally, some VAs are associated with germline variants in the RAS pathway (so called RASopathies), PTEN, and TEK, among other genes [8, 15]. 

Accurate diagnosis and management of VAs is challenging, and these patients often receive incorrect diagnoses and inappropriate treatments. For example, one study found that nearly half of patients presenting to VA clinics with complex VAs had incorrect diagnoses [16]. These patients also experience challenges with communication due to a lack of doctors with knowledge about VAs [17]. 

In April 2022, the US Food and Drug Administration (FDA) approved alpelisib for the treatment of certain VAs with severe manifestations in patients older than 2 years of age. Growing evidence also supports the use of other targeted inhibitors for the treatment of VAs, often in combination with other modalities such as sclerotherapy, surgery, or laser ablation [18–24]. Given the continued rapid growth in knowledge and availability of treatments for VAs, it is essential that patients with complex VAs can access expert, coordinated care from knowledgeable specialists.

Previous qualitative research shows that patients with VAs experience major barriers to accessing multidisciplinary care with VA experts including insurance obstacles, care coordination challenges, and long distances to multidisciplinary clinics [25], given that multidisciplinary VA centers are scarce in many regions of the US [26]. Moreover, few clinicians are familiar with VAs due to the rarity of these disorders and complex presentations of symptoms, leading to inaccurate diagnoses, inappropriate treatments, worsened health, and delayed referrals to VA experts [27, 28]. VA specialists also typically focus on pediatric care, meaning adult patients lack clinicians with expertise or interest in VAs [29]. As a result, parents of patients with VAs, and adult patients with VAs must relentlessly self-advocate to receive needed care and take on complex care coordination tasks [25]. Given the potential risks of delayed or inaccurate treatments, we must determine which factors facilitate or impede the ability of patients with VAs to navigate the healthcare system in order to receive expert VA care.

The ability to navigate the healthcare system is an important dimension of communicative health literacy that can significantly impact patient self-management and health outcomes [30, 31]. Therefore, in this survey study, we aimed to identify factors associated with adult patients’ self-reported ability to navigate the healthcare system. Guided by previous research, we hypothesized that the ability to navigate the healthcare system would be associated with the quality of information exchange with clinicians (H_1_), patient self-advocacy (H_2_), and whether the patient receives care from a VA specialist or team of specialists (H_3_). Given that many patients also experience high levels of emotional distress and anxiety [32], we also hypothesized that the ability to navigate the healthcare system would be significantly associated with anxiety (H_4_). To explore potential sociodemographic factors, we hypothesized that age, race and ethnicity, gender, and level of education would be significantly associated with the ability to navigate the healthcare system (H_5_).

## Results

### Participant characteristics

Of 166 total responses, 27 were excluded from this analysis due to missing values for demographic information, yielding a final analytic cohort of 139 participants for bivariable analyses where we identified the association between participants’ ability to navigate the healthcare system, and quality of information exchange, self-advocacy, specialist care, anxiety, and socio-demographic factors. Due to missing values for included variables, we excluded another 3 responses in our multivariable analyses, where we predicted participants’ ability to navigate the healthcare system in a single model including all above covariates. Participants spent a median of 24 min completing the survey (interquartile range = 16 to 36 min). Participants were predominantly White and non-Hispanic (*n* = 109, 79%), and 73% of participants had earned a college or graduate degree. The majority of participants had been diagnosed with a phenotype of PIK3CA-Related Overgrowth Spectrum (*n* = 107, 78%). Half of the participants were being cared for by a VA specialist or team of specialists, and these participants lived a median of 20 miles from their VA specialists (Table 1).

**Table 1:** Participant characteristics

Characteristic	n	%
Age in Years– Mean (SD)	41.4	(14.3)
Gender^a^		
Woman	90	(66%)
Man	45	(33%)
Non-binary/third gender	1	(1%)
Race^b^		
American Indian or Alaska Native	2	1%
Asian	8	6%
Black or African American	7	5%
Native Hawaiian or Pacific Islander	1	1%
White	127	91%
Hispanic, Latino, or Spanish Ethnicity	14	10%
Language Spoken at Home		
English	129	93%
Spanish	4	3%
Other	6	4%
Education		
Some High School	5	4%
High School or Equivalent	11	8%
Some College	22	16%
College Degree	57	41%
Graduate/Professional Degree	44	32%
Household Income^c^		
$24,999 or less	7	5%
$25,000 - $49,999	22	16%
$50,000 - $74,999	20	15%
$75,000 - $99,999	21	15%
$100,000 or greater	44	32%
Relationship Status		
Married or Living as Married	65	47%
Never Married	51	37%
Divorced/Separated	18	13%
Widowed	4	3%
Residential Status^d^		
Metropolitan	92	90%
Micropolitan	5	5%
Small Town/Rural	5	5%
Distance from Vascular Anomaly Specialist (median, Interquartile Range)	20 miles	(8.8, 151)
Have a primary care doctor	115	83%
Have a VA specialist or team of specialists	69	50%
Diagnosis^e^		
PIK3CA-Related Overgrowth Spectrum	107	78%
CLOVES Syndrome	13	9%
Fibro-Adipose Vascular Anomaly	11	8%
Klippel-Trenaunay Syndrome	76	55%
Gorham Stout Disease	2	1%
Generalized Lymphangiomatosis	5	4%
Central Conducting Lymphatic Anomaly	2	1%
PHACE Syndrome	2	1%
Tufted Angioma	1	< 1%
Parkes Weber	2	1%
Other	4	3%
Not Diagnosed with Syndrome	14	10%

^a^2 participants preferred not to describe their gender; ^b^Racial categories not mutually exclusive, and 7 participants reported multiple races; ^c^ 20 participants preferred not to characterize their income; ^d^Restricted to US residents, based on RUCA codes determined by zip code. 24 participants resided in countries outside of the US, and 13 participants declined to provide their zip code.^e^ Many of these disorders are often grouped in the larger diagnostic category of PIK3CA-Related Overgrowth Spectrum (PROS). However, many patients self-reported their disorders by these historic terms, rather than as PROS. These percentages reflect patients self-reporting, which we aggregated into the PROS category

### Participant experiences with navigating the healthcare system

Most participants reported that navigating the healthcare system was “sometimes” or “usually difficult” (mean score 16.4/30, standard deviation (SD) 5.6). Participants also reported high anxiety (T-score 58, SD 8.9), and high levels of self-advocacy (43.5/57). Summed Information Exchange scores (21.8/30) equated to an average response between “sometimes” and “often” for the frequency of each information exchange behavior (Table 2).

**Table 2:** Participants’ experiences with vascular malformations

	Sample Means (SD)
**Dependent Variable**	
Ability to Navigate the Healthcare System^a^	Mean Total Score = 16.4 (5.6)
**Independent Variables**	
Quality of Information Exchange^b^	Mean Total Score = 21.8 (5.6)
Patient Self-Advocacy^c^	Mean Total Score = 43.5 (6.2)
Anxiety^d^	Mean T-Score = 58.0 (8.9)
Clinician Knowledge (Target Efficacy)^e^	Mean Total Score = 10.8 (4.3)

^a^HLQ “ability to navigate healthcare” subscale possible total scores range from 6 to 30

^b^PCC-Ca-36 “information exchange” subscale possible total scores range from 6 to 30

^c^PSAS possible total scores range from 12 to 60

^d^PROMIS Anxiety was transformed to a T-score, where the general population mean is T = 50

^e^The TMIM “target efficacy” subscale possible total scores range from 4 to 20

### Factors associated with ability to navigate the healthcare system

In bivariate linear regression, patients who reported a higher ability to navigate the healthcare system were also more likely to report higher quality of information exchange (β = 0.44, 95% Confidence Interval (CI) 0.28 to 0.59, *p* <.001) and have a VA specialist (β = 2.86, 95% CI 1.04 to 4.68, *p* =.002). Age also had a statistically significant, but small, negative association (β=-0.07, 95% CI − 0.14 to − 0.01, *p* <.034) with the ability to navigate the health care system. For participants who had a primary care doctor (*n* = 114), ratings of the primary care doctor’s VA knowledge were positively associated with the ability to navigate the healthcare system (β = 0.37, 95% CI 0.15 to 0.60, *p* =.001) (Table 3).

**Table 3:** Bivariate linear regression models

	Ability to Navigate Healthcare System	
	β (95% CI)	*P*
**Quality of Information Exchange**	**0.44 (0.28 to 0.59)**	**< 0.001**
Patient Self-Advocacy	0.02 (-0.13 to 0.18)	0.763
Anxiety	− 0.09 (-0.19 to 0.02)	0.110
Have a Primary Care Doctor	− 0.32 (-2.81 to 2.17)	0.800
**Have a VA Specialist or Specialist Team**	**2.86 (1.04 to 4.68)**	**0.002**
**Knowledgeable Primary Care Doctor**	**0.37 (0.15 to 0.60)**	**0.001**
College Degree (Ref: No College Degree)	-1.91 (-3.99 to 0.18)	0.073
**Age**	**− 0.07 (-0.14 to − 0.01)**	**0.034**
Non-Hispanic White (Ref: All other Races/Ethnicities)	− 0.14 (-2.45 to 2.17)	0.902
Female (Ref: Male or Non-Binary)	-1.00 (-2.60 to 0.60)	0.219
Annual Household Income*	0.02 (-0.47 to 0.51)	0.942

*n = 118 for annual household income, because 20 participants ‘preferred not to answer’

The results of multivariate analysis were similar: the ability to navigate the healthcare system was positively associated with quality of information exchange (β = 0.38, 95% CI 0.22 to 0.55, *p* <.001) and whether patients had a VA specialist (β = 2.31, 95% CI 0.35 to 4.28, *p* =.021), but not associated with patient self-advocacy, anxiety, education, age, race and ethnicity, gender, or having a primary care physician (Table 4). For participants with primary care doctors, the ability to navigate the healthcare system was positively associated with quality of information exchange (β = 0.27, 95% CI 0.09 to 0.45, *p* =.004), having a VA specialist (β = 2.31, 95% CI 0.22 to 4.39, *p* =.031), and primary care doctors’ level of VA knowledge (β = 0.27, 95% CI 0.04 to 0.50, *p* =.023) (Table 5).

**Table 4:** Multivariate linear regression model (*n* = 136)

	Ability to Navigate Healthcare System	
	β (95% CI)	*P*
**Quality of Information Exchange**	**0.38 (0.22 to 0.55)**	**< 0.001**
Patient Self-Advocacy	− 0.03 (-0.18 to 0.11)	0.674
Anxiety	− 0.08 (-0.18 to 0.03)	0.136
Have a Primary Care Physician	− 0.55 (-3.05 to 1.95)	0.664
**Have a VA Specialist or Specialist Team**	**2.31 (0.35 to 4.28)**	**0.021**
College Degree (Ref: No College Degree)	-1.48 (-3.56 to 0.59)	0.159
Age	0.02 (-0.05 to 0.09)	0.551
Non-Hispanic White (Ref: All other Races/Ethnicities)	− 0.47 (-2.72 to 1.78)	0.681
Female (Ref: Male or Non-Binary)	− 0.82 (-2.31 to 0.68)	0.282

**Table 5:** Exploratory analysis of primary care doctor’s knowledge of VAs (*n* = 114)*

	Ability to Navigate Healthcare System	
	β (95% CI)	*P*
**Quality of Information Exchange**	**0.27 (0.09 to 0.45)**	**0.004**
Patient Self-Advocacy	− 0.03 (-0.18 to 0.12)	0.738
Anxiety	− 0.10 (-0.20 to 0.00)	0.060
**Knowledgeable Primary Care Doctor**	**0.30 (0.07 to 0.52)**	**0.011**
**Have a VA Specialist or Specialist Team**	**2.31 (0.216 to 4.39)**	**0.031**
College Degree (Ref: No College Degree)	-1.10 (-3.12 to 1.23)	0.352
Age	0.04 (-0.04 to 0.11)	0.341
Non-Hispanic White (Ref: All other Races/Ethnicities)	− 0.06 (-2.56 to 2.45)	0.965
Female (Ref: Male or Non-Binary)	− 0.76 (-2.41 to 0.90)	0.367

*24 participants did not have a primary care doctor. 1 participant had missing data for their primary care doctor’s level of VA knowledge

## Discussion

Our findings add to a growing literature that documents multiple barriers to receiving expert care for patients with rare diseases [33–36]. These results also reaffirm our prior qualitative results demonstrating that families affected by vascular anomalies experience multiple and enduring barriers to expert care and adequacy of information influenced each of these factors [25]. Furthermore, our findings suggest an important role for primary care doctors in helping patients to navigate the healthcare system. Despite high levels of self-advocacy, our findings also suggest that patients need additional supports and systemic changes to healthcare system delivery in order to improve their ability to receive necessary medical care.

Participants who reported a higher quality of information exchange with their physicians were more likely to also report a greater ability to navigate the healthcare system. This association persisted after adjusting for whether patients accessed specialist VA care and the level of their primary care doctor’s VA knowledge. This finding suggests that high-quality information could improve the ability of patients with VAs to access needed care. Prior work in other rare diseases has also described the important role of information. For example, a population-based study of rare disease care in China found that patients who had more difficulty finding high-quality information on rare disease management were more likely to experience misdiagnosis [37]. When clinicians fail to provide high-quality information, patients with VAs rely on the internet and social media sources, similar to other rare diseases [17, 38, 39]. The quality of information from these sources varies, and patients could be exposed to incorrect or misleading information [40]. To support patients as they advocate for their care, clinicians and researchers should strive to develop and disseminate reliable sources of information for patients with VAs. This could include patient-friendly pamphlets, understandable images, and videos that are co-developed with patient advocates. Academic institutions could consider the development of these materials as scholarly contributions that support career advancement. Furthermore, funding agencies could prioritize open-access publications and lay person summaries of research findings.

In our exploratory analysis, patients who reported having more knowledgeable primary care doctors also reported a greater ability to navigate the healthcare system. However, many patients report that primary care doctors lack knowledge of VAs [25]. In total, more than 6000 rare diseases have been described, and VAs represent only a small fraction of these diseases [41]. Unsurprisingly, physicians often lack knowledge about rare diseases [42–44] and medical curricula lack education about most rare diseases [45, 46]. This finding supports the need for increased physician education of VAs. Unknowledgeable physicians may provide inadequate information to patients, and patients may avoid communicating with clinicians they perceive to be inadequate sources of illness-related information [37]. However, while we agree that there is a need for more education and training on managing care for patients with rare diseases in general, this type of education will not provide physicians with up-to-date knowledge about each of the 6000 rare disorders they might encounter in clinical practice. As such, future research should identify what educational interventions are ideal for ensuring clinicians who lack VA expertise receive just-in-time education when they encounter patients with VAs. Our previous work also demonstrates the importance of physicians showing a commitment to doing research and learning more when they encounter a patient with a VA [33]. As such, physicians who are unfamiliar with VAs should demonstrate an ongoing commitment to staying up-to-date on the evolving literature or connecting with VA specialists.

While patients reported a high level of self-advocacy, self-advocacy was not associated with a better ability to navigate the healthcare system. This finding is surprising, given the central role of self-advocacy in VA care shown in prior studies [25, 47], but suggests that even the most motivated and persevering patients need support when navigating the system of care for VAs. For example, previous research suggests that patients who live far from VA centers might encounter financial or insurance issues that make access more difficult regardless of how tirelessly they advocate for care [25]. Additionally, patients who are unable to find knowledgeable clinicians or obtain reliable information on VAs might not know how to best advocate for themselves. To ensure high-quality care for patients with VAs, healthcare systems and policymakers must strive to make systemic improvements to the healthcare system rather than relying on patients to navigate the healthcare system without the necessary support. These systemic changes could include improved collaborations between specialists and primary care physicians, increased utilization of telehealth technologies to support financially disadvantaged or rural-residing families, and decision support tools to help non-specialist clinicians care for patients who are unable to access care from VA specialists.

Finally, in our study age, education, sex, and race/ethnicity did not seem to play a significant role in participants’ ability to navigate the healthcare system. In the bivariate model, age was significantly associated with the ability to navigate care; however, this association was no longer significant in the multivariate model. While this finding suggests that older patients may need additional assistance navigating care, high-quality information exchange and access to VA specialists are important for all patients regardless of age.

Our results should be interpreted in light of limitations. First, despite sampling from multiple VA support groups and VA multidisciplinary clinics, our sample was predominantly White, well-educated participants residing in metropolitan areas. We were unable to analyze the role of being from a historically marginalized group, having less education, and living in a rural area in our regression models due to the small proportion of participants from these groups. As such, our results might underrepresent the barriers experienced by these groups of patients, who are likely to experience additional systemic barriers to navigating the healthcare system. These skewed demographics could be related to our study design, or they could be related to underlying disparities in which patients are able to get a diagnosis and access expert, multidisciplinary care. Future studies should evaluate the sociodemographic characteristics of patients who are able to access expert care in order to identify potential disparities in diagnosis rates. Furthermore, our sample size limited the number of variables we were able to include in our regression models. It is possible that other important participant characteristics or experiences might influence the ability of VA patients to navigate the healthcare system. Lastly, these associations are not necessarily indicative of causation. As such, it is possible that patients who are able to better navigate the healthcare system are also more likely to reach more knowledgeable doctors and receive better information. However, this association between navigating the healthcare system and information exchange persisted even when adjusting for whether patients have a VA specialist and their primary care doctor’s level of VA knowledge. This finding does not prove causation, but makes it plausible that information exchange is supporting these patients’ ability to navigate the healthcare system.

## Conclusion

Patients with VAs struggle to navigate the healthcare system and access necessary medical care. High-quality information from clinicians and more knowledgeable primary care doctors might help patients to access VA care. Relying on patient self-advocacy is insufficient to improve healthcare access for patients with VAs. Future efforts should focus on patient-directed and clinician-directed educational interventions. Additionally, future work should assess the structural barriers that impede healthcare access for these patients.

## Methods

### Participants and recruitment

We surveyed adult patients with VAs, recruiting participants from patient advocacy groups and multidisciplinary vascular anomaly clinics. We collaborated with five patient advocacy groups that provide support for families affected by lymphatic and vascular anomalies to disseminate a survey flyer through their online and social media platforms: Klippel-Trenaunay Support Group, CLOVES Syndrome Community, Project FAVA, Lymphangiomatosis and Gorham’s Disease Alliance, and PHACE Syndrome Community. We also distributed flyers via email to vascular anomaly specialists at every multidisciplinary vascular anomaly center in the US. Our goal in recruiting from patient advocacy groups and VA clinics was to reach potential participants with diverse sociodemographic characteristics and diverse experiences in navigating the healthcare system. Eligibility criteria included (1) 18 years or older and (2) self-reported vascular anomaly diagnosis. Patients who were interested in the study followed a URL or QR code to a study webpage that confirmed their eligibility. Interested eligible participants provided anonymous responses online using Qualtrics survey software. We recruited participants between June 2022 and October 2022. Participants were offered $25 electronic gift cards after completing the survey.

### Data collection

Questionnaires included previously validated measures, select new items focused on experiences with emergency care, and demographic questions. The primary outcome was the perceived ability to navigate the healthcare system using the “Navigating the Healthcare System” subscale of the Health Literacy Questionnaire (HLQ) [30]. This six-item measure evaluates a patient’s ability to find the right healthcare, know what types of services are needed and available, and access these healthcare resources.

To test the hypothesis that the ability to navigate the healthcare system is associated with the quality of information exchange (H_1_), self-advocacy (H_2_), specialist team composition (H_3_), and anxiety (H_4_), we examined several measures from our survey. We evaluated the quality of information exchange using the five-item “Exchanging Information” subscale of the Patient-Centered Communication in Cancer (PCC-Ca-36) measure [48]. We assessed patient self-advocacy using the 12-item Patient Self-Advocacy Scale [49]. We assessed anxiety with the four-item PROMIS Short Form v1.0 - Anxiety − 4a. We assessed the primary care doctor’s knowledge of VAs using an adapted version of the Target Efficacy Subscale of the Theory of Motivated Information Management instrument developed by Fowler and Afifi [50]. The survey included additional measures not included in the current analysis: remaining HLQ and PCC-Ca-36 subscales, PROMIS Short Form v1.0 - Self-Efficacy for Managing Symptoms − 8a, Trust in Physician Scale [51], PROMIS Scale v1.1– Global Health Scale, and PROMIS Short Forma v1.0– Depression– 4a. We also asked questions about experiences with emergency care, and open-ended questions about best and worst experiences.

Patients were asked to report their gender, age, race, ethnicity, language spoken at home, highest level of education, annual household income, marital status, zip code, country of residence, distance from VA specialist, and diagnosis. We also asked whether participants had a primary care doctor and which kind of doctor provided most care for their VA. This study was approved by the institutional review board at Washington University. Data were stored in an institutional, encrypted cloud-based server. Participant names were removed from the dataset and replaced with study ID numbers. We maintained a separate document that linked these participant names to study IDs.

### Statistical analysis

The Navigating the Healthcare System, Exchanging Information, Patient Self-Advocacy and Knowledgeable Primary Care Doctor scores were calculated by summing responses from items of each scale. The anxiety score was calculated by summing the responses from 4 items and converting these summative scores to T-scores using T-score tables provided with the PROMIS measure. With this conversion to T-scores, a score of 50 is the mean of a relevant reference population, and 10 is the standard deviation of that population. The minimal important change for PROMIS measures ranges between 2 and 6 T-score points [52]. 

Next, we performed a series of linear regressions to test our hypotheses. We hypothesized that the ability to navigate the healthcare system would be associated with: (a) quality of information exchange, (b) patient self-advocacy, (c) anxiety, and (d) whether the patient has a VA specialist. We also hypothesized that age, race and ethnicity, gender, and level of education were important to include as variables. For education, we dichotomized at achieving a college degree. Due to the limited diversity of this sample, we dichotomized race into two categories: White, Non-Hispanic participants and all other races and ethnicities. First we performed bivariate linear regression with ability to navigate the healthcare system as the dependent variable. We then performed multivariate linear regression with the ability to navigate the healthcare system as the dependent variable, forcing entry of these three scale scores and four demographic variables in a single model. To assess for multicollinearity, we calculated the variance inflation factor (VIF). All VIFs were < 2, indicating low likelihood of multicollinearity. As an exploratory analysis of participants who had a primary care doctor, we tested another multivariate linear regression model that also included a measure of the primary care doctor’s level of VA knowledge. All analyses were performed in IBM SPSS statistical package v28.0.0.0.

## Data Availability

The raw data generated and/or analyzed during the current study are not publicly available due ethical restrictions related to ensuring confidentiality, but de-identified data are available from the corresponding author on reasonable request.

## Declarations

### Authors’ information

BAS is a pediatric oncology physician specializing in VMs; AK is a medical educator with a PhD in communication. Both authors had led multiple prior qualitative and quantitative studies. SL is a public health research with expertise in systems-level approaches to improving healthcare delivery, and training and experience in biostatistics.

## Abbreviations

VA: Vascular anomaly
CLOVES: Congenital Lipomatous Overgrowth, Vascular Malformations, Epidermal Nevis, Skeletal anomalies
HLQ: Health Literacy Questionnaire
PROMIS: Patient-Reported Outcomes Measurement Information System
